# Decompensated heart failure

**DOI:** 10.1590/S1679-45082013000300022

**Published:** 2013

**Authors:** Sandrigo Mangini, Philippe Vieira Pires, Fabiana Goulart Marcondes Braga, Fernando Bacal

**Affiliations:** 1 Hospital Israelita Albert Einstein, São Paulo, SP, Brazil;; Hospital das Clínicas, Faculdade de Medicina, Universidade de São Paulo, São Paulo, SP, Brazil.; 2 Hospital Israelita Albert Einstein, São Paulo, SP, Brazil.; 3 Hospital das Clínicas, Faculdade de Medicina, Universidade de São Paulo, São Paulo, SP, Brazil.

**Keywords:** Heart failure/diagnosis, Heart failure/therapy, Prognosis

## Abstract

Heart failure is a disease with high incidence and prevalence in the population. The costs with hospitalization for decompensated heart failure reach approximately 60% of the total cost with heart failure treatment, and mortality during hospitalization varies according to the studied population, and could achieve values of 10%. In patients with decompensated heart failure, history and physical examination are of great value for the diagnosis of the syndrome, and also can help the physician to identify the beginning of symptoms, and give information about etiology, causes and prognosis of the disease. The initial objective of decompensated heart failure treatment is the hemodynamic and symptomatic improvement preservation and/or improvement of renal function, prevention of myocardial damage, modulation of the neurohormonal and/or inflammatory activation and control of comorbidities that can cause or contribute to progression of the syndrome. According to the clinical-hemodynamic profile, it is possible to establish a rational for the treatment of decompensated heart failure, individualizing the proceedings to be held, leading to reduction in the period of hospitalization and consequently reducing overall mortality.

## RESUMO

A insuficiência cardíaca apresenta elevada incidência e prevalência em todo mundo. Os custos com internação por insuficiência cardíaca descompensada chegam a aproximadamente 60% do custo total do tratamento da insuficiência cardíaca, e a mortalidade durante a internação varia conforme a população estudada, podendo chegar a 10%. Em pacientes com insuficiência cardíaca descompensada, os achados de história e exame físico são de grande valor por fornecerem, além do diagnóstico da síndrome, o tempo de início dos sintomas, as informações sobre etiologia, as causas de descompensação e o prognóstico. O objetivo inicial do tratamento da insuficiência cardíaca descompensada é a melhora hemodinâmica e sintomática. Além disso, outros alvos devem ser buscados, incluindo preservação e/ou melhora da função renal, prevenção de lesão miocárdica, modulação da ativação neuro-hormonal e/ou inflamatória, e manejo de comorbidades que podem causar ou contribuir para progressão da síndrome. Com base nos perfis clínico-hemodinâmicos, é possível estabelecer um racional para o tratamento da insuficiência cardíaca descompensada, individualizando o procedimento a ser instituído e objetivando redução de tempo de internação e de mortalidade.

## INTRODUCTION

Decompensated heart failure (DHF) is defined as a clinical syndrome in which a structural or functional change in the heart leads to its inability to eject and/or accommodate blood within physiological pressure levels, thus causing a functional limitation and requiring immediate therapeutic intervention^(1)^. It has an irrefutable epidemiological importance, and clinical peculiarities that directly influence treatment. The objective of this study is to guide clinicians on the current management of DHF.

## EPIDEMIOLOGY

HF has a high incidence and prevalence worldwide. One to two percent of the population of developed countries are estimated to have HF, and this prevalence increases to 10% in the population 70 years of age or over. In Europe, 10 million people are estimated to have HF with associated ventricular dysfunction, and other 10 million, to have HF with preserved ejection fraction (HFPEF)^(2,3)^. Brazilian 2012 data demonstrated that 21.5% of 1,137,572 hospitalizations for diseases of the circulatory system were for HF, with a 9.5% in-hospital mortality, and 70% of the cases in the age range above 60 years^(4)^.

Costs with hospitalizations for decompensation reach approximately 60% of the total expenditures with the treatment of HF^(5)^. Mortality rate among patients discharged within 90 days is of approximately 10%, with roughly 25% of readmissions in the period^(5)^.

Ischemic cardiomyopathy is considered the most common cause of HF^(6)^. However, in Brazil, hypertensive, chagasic, and valvular cardiomyopathies play an important role, including in relation to hospitalizations for decompensation^(7,8)^.

## CLASSIFICATION OF DECOMPENSATED HEART FAILURE

DHF may present in the acute form or as an acute exacerbation of chronic HF, and may be classified as follows^(8)^.

### “New” acute HF (not previously diagnosed)

Clinical HF syndrome which occurs in patients with no previous signs and symptoms of HF, triggered by clinical situations such as acute myocardial infarction, hypertensive crisis, and rupture of the mitral chordae tendineae. In this context, pulmonary congestion is usually present without systemic congestion, and blood volume is generally normal. The use of high doses of diuretics is not indicated, but rather treatment of the primary cause of decompensation (vasodilator in hypertensive crisis, artery opening in acute coronary syndrome – ACS, and correction of mitral regurgitation in rupture of the chordae tendineae).

### Decompensated chronic HF (acute exacerbation of chronic HF)

Clinical situation in which there is acute or gradual exacerbation of signs and symptoms of HF at rest in patients previously diagnosed with HF, that requires additional and immediate therapy. This is the most frequent clinical presentation of DHF^(8)^, and its most common cause is low treatment adherence (water and sodium restriction and inadequate use of medications). Other important causes include: infection, pulmonary embolism, use of medications such as antiinflammatory drugs, and tachy- or bradiarrhythmias. It is usually related to pulmonary and/or systemic congestion, with evident hypervolemia. In addition to seeking the cause of decompensation, volume management with diuretics is crucial.

## CLINICAL PRESENTATION

In patients with DHF, findings from history taking and physical examination are important not only for providing the diagnosis of the syndrome, but also the time of onset of symptoms, information on the etiology, causes of decompensation (Chart 1) and prognosis.

**Chart 1:** Triggering factors of decompensation in heart failure

Excessive water and salt intake	
Non-adherence to treatment and/or lack of access to medication	
Excessive physical exertion	
Acute atrial fibrillation or other tachyarrhythmias	
Bradyarrhythmias	
Systemic hypertension	
Pulmonary thromboembolism	
Myocardial ischemia	
Fever and infections	
Elevated room temperature	
Anemia, nutritional deficiencies, AV fistulas, thyroid dysfunction, decompensated diabetes	
Excessive alcohol consumption	
Renal failure	
Pregnancy	
Depression	
Use of illicit drugs (cocaine, crack, ecstasy, and others)	
Social factors (abandonment, social isolation)	
Inappropriate prescription or at insufficient doses (different from those recommended in guidelines)	
Factors related to physicians	
	Lack of training in the management of patients with HF
	Failure to provide adequate patient advice in relation to diet and physical activity
	Undetected volume overload (lack of daily weight control)
	IV fluid overload during hospitalization
Factors related to medications	
	Digitalis intoxication
	Water-retaining or prostaglandin-inhibiting drugs: NSAIDs, steroids, estrogens, androgens, chlorpropamide, glitazones, minoxidil
	Negative inotropic drugs: group I antiarrhythmic drugs, calcium channel antagonists (except amlodipine), tricyclic antidepressants
	Drugs toxic to the myocardium: cytostatic drugs such as adriamycin
	Self-medication, alternative therapies

Source: Bocchi et al^(1)^.

AV: arteriovenous; IV: intravenously; NSAIDs: non-steroidal antiinflammatory drugs; HF: heart failure.

The most common and characteristic symptom of DHF is dyspnea. However, this finding has low specificity, and may be found in other clinical conditions. This is also true for the presence of nocturnal cough, leg edema, pulmonary wheezes or rales. On the other hand, orthopnea, paroxysmal nocturnal dyspnea, and presence of the third heart sound, although not pathognomonic, are more specific signs and symptoms of HF^(9)^. Personal health and family history, as well as the review of systems, may add data to infer the etiology and presence of comorbidities.

Identifying the cause is important, since it can help to choose specific therapies (myocardial revascularization in ischemic cardiomyopathy), to infer the prognosis (greater severity of chagasic and ischemic cardiomyopathies)^(1,7)^, and to guide the pharmacological treatment of decompensation.

Based on the findings of bedside physical examination, it is possible to define the clinical-hemodynamic profile with the purpose of guiding treatment of DHF, as well as stratifying its risk using congestion and perfusion parameters. The presence of congestion can be inferred in 70% to 80% of DHF cases, by means of signs of tachypnea, pulmonary crackles, third heart sound, increased jugular venous pressure, leg edema, tender hepatomegaly, hepatojugular reflux, pleural effusion and ascites. The presence of poor perfusion is related to the findings of tachypnea, hypotension, pulsus alternans, prolonged capillary filling time, cyanosis, and abnormal level of consciousness.

**Figure 1:**
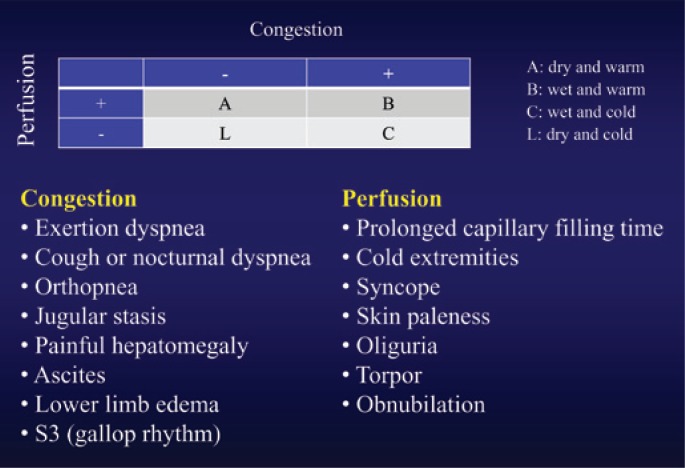
Clinical assessment of decompensated heart failure

According to the algorithm developed by Stevenson^(10)^, patients presenting with congestion are classified as “wet”, whereas patients without congestion are called “dry”. Patients with inadequate perfusion are classified as “cold”, whereas those with good perfusion are classified as “warm”. Thus, four clinical-hemodynamic profiles are defined (Figure 1): profile A (“dry-warm” or compensated); profile B (“wet-warm”, which is the most common type); profile C (“wet-cold”, with the worst prognosis); and profile L (“dry-cold”, which is not frequent).

## DIAGNOSTIC STUDIES IN DECOMPENSATED HEART FAILURE

**Figure 2:**
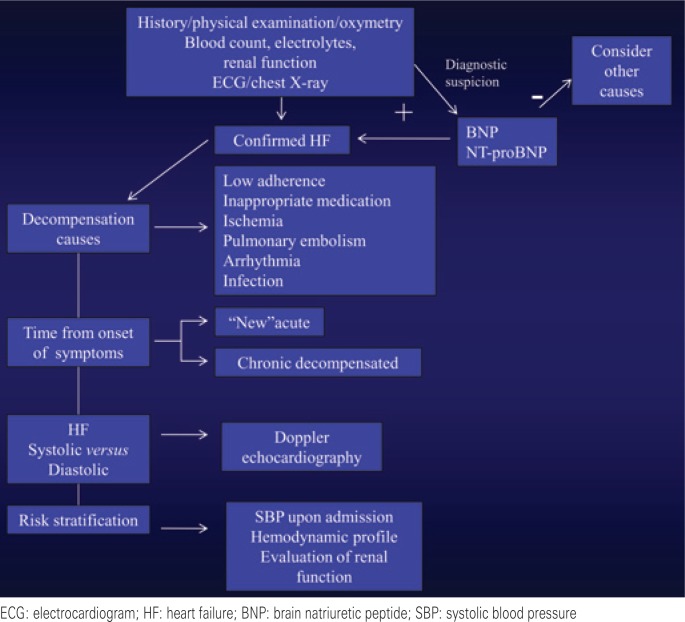
Diagnostic assessment of decompensated heart failure

Although the diagnosis of DHF is made based on data from history and physical examination, diagnostic studies are important because, in addition to confirming the diagnosis, they also provide data on the degree of cardiac remodeling, the presence of systolic and/or diastolic dysfunction, etiology, cause of decompensation, presence of comorbidities, and risk stratification (Figure 2). Among the diagnostic studies available, the following are specially helpful.

### Electrocardiography

Fundamental in the management of ACS. Some findings may suggest specific etiologies: the presence of Q waves, absence of R wave progression in precordial leads and repolarization abnormalities, especially of the ST-segment, suggest an ischemic component; the association of right bundle branch block with left anterior superior division block suggests Chagas disease; low voltage in the frontal plane suggests storage disease and pericardial effusion. The presence of left bundle branch block may correspond to acute myocardial infarction or pronounced myocardial remodeling, thus characterizing a poor prognosis. Bradyarrhythmias and tachyarrhythmias may be the cause of DHF, and have therapeutic and prognostic implications.

### Laboratory tests

Blood count, BUN, creatinine, blood glucose, electrolytes, and urinalysis are simple methods that help define comorbidities, the cause of decompensation, prognosis and treatment. When ACS is suspected, myocardial necrosis markers are important for the diagnosis; also, increased levels in the absence of obstructive coronary disease have a prognostic value. Arterial blood gases, central venous blood gases, lactate, and tests to check liver integrity and function should be performed in more severely ill patients. Thyroid profile and serologic test for Chagas disease may be considered.

### Biomarkers

Biomarkers are useful in the diagnosis and prognosis of DHF. Among the several biomarkers that have been studied, natriuretic peptides, BNP and NT-ProBNP are the most widely used and well established in the clinical practice. They are produced mainly in the ventricles, in response to increased ventricular wall tension. Determination of their levels is indicated for the differential diagnosis of dyspnea in the emergency room^(11,12)^. Increased levels are found in systolic dysfunction and in HFPEF (greater levels in systolic dysfunction). They have prognostic value and have been considered markers of response to the treatment of DHF, despite controversial findings^(13,14)^. Recently, a Brazilian study demonstrated a diagnostic and prognostic impact of exhaled acetone in DHF^(15)^.

### Echocardiography

This is the main noninvasive method for the diagnosis of HF. In patients with DHF, it is indicated to help find the etiology and establish the prognosis, in addition to give information on the type of dysfunction (systolic and/or diastolic), chambers affected, heart valve lesions, segmental contractility abnormalities and pericardium. In DHF, it may show the progression of dysfunction and the cause of decompensation (pericardial effusion, pulmonary embolism, and acute ischemia). It also may be used for the definition of the hemodynamic profile and to guide therapy (hemodynamic echo)^(16)^.

### Pulmonary artery catheter

It permits the direct analysis of intracardiac and intravascular pressures, as well as of microhemodynamics parameters. It is indicated to help treat patients with DHF, especially in the presence of shock and for the assessment of the pulmonary vascular resistance, to indicate cardiac transplantation. The ESCAPE study did not show benefit of the use of a pulmonary artery catheter in the treatment of DHF without cardiogenic shock^(17)^.

## RISK STRATIFICATION IN DECOMPENSATED HEART FAILURE

As shown in chart 2, there is a series of poor-prognosis factors related to DHF^(1)^. Among those mentioned, we should point out systolic blood pressure (SBP) and renal function (ADHERE registry)^(18)^. Patients with DHF presenting BUN>90mg/dL, SBP<115mmHg and creatinine>2.75mg/dL on admission have a 21.9% risk of in-hospital mortality; on the other hand, patients not presenting these characteristics have a low mortality risk (2.14%). In DHF, the cardiorenal syndrome is related to different mechanisms, in special, renal hypoperfusion due to myocardial dysfunction or hypovolemia and systemic congestion with renal venous hypertension^(19)^. An increase by 0.3mg/dL in creatinine levels on admission is related to higher mortality^(20)^.

**Chart 2:** Factors of worse prognosis in decompensated heart failure

Age (above 65 years)
Hyponatremia (sodium <130meq/L)
Impaired renal function
Anemia (hemoglobin <11g/dL)
Signs of peripheral hypoperfusion
Cachexia
Complete left bundle branch block
Atrial fibrillation
Restrictive pattern on Doppler
Persistent elevation of natriuretic peptides levels despite treatment
Persistent congestion
Persistent third heart sound
Sustained ventricular tachycardia or ventricular fibrillation

Source: Bocchi et al^(1)^.

DHF: decompensated heart failure.

## TREATMENT OF DECOMPENSATED HEART FAILURE

The initial objective of the treatment of DHF is to achieve hemodynamic and symptomatic improvement. In addition, other targets should be sought, including the preservation and/or improvement of the renal function, prevention of myocardial damage, modulation of the neurohormonal and/or inflammatory activation, and management of comorbidities that could cause or contribute to the progression of the syndrome^(21)^.

**Figure 3:**
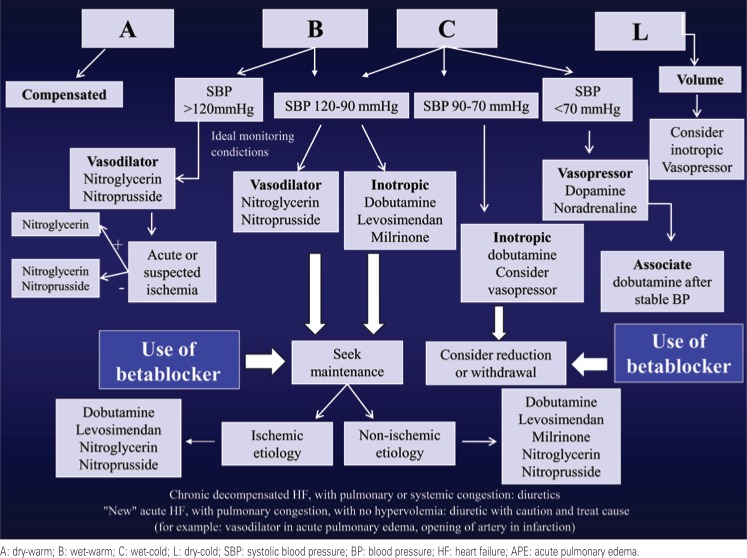
Treatment algorithm of decompensated heart failure

Based on the hemodynamic profiles proposed by Stevenson, on the assessment of volemia, on the definition of the time of onset of symptoms, on the cause of decompensation, and on the SBP, it is possible to establish a rationale for the treatment of DHF (Figure 3).

Most of the patients with decompensation show a predominance of pulmonary and/or systemic congestion and adequate peripheral perfusion (“wet-warm” pattern – profile B), and their treatment include vasodilators and diuretics. If worsening of the renal function occurs, inotropic drugs should be considered (especially when the SBP is between 90 and 120mmHg). In situations of congestion and poor peripheral perfusion (“wet-cold” pattern – profile C), inotropic drugs and diuretics are indicated; if BP is being intensively monitored, it is also possible to use intravenous vasodilators. The finding of poor perfusion without pulmonary congestion is rare (“dry-cold” pattern – profile L) and usually responds to volume (inotropic drugs may be necessary).

Criteria for hospitalization are shown in Chart 3 ^(1)^.

**Chart 3:** Criteria for hospitalization

Criteria for immediate hospitalization	Pulmonary edema or respiratory distress in the sitting position
	Oxygen saturation <90%
	Heart rate >120bpm in the absence of chronic atrial fibrillation
	Systolic blood pressure <75mmHg
	Mental disorder attributable to hypoperfusion
	Decompensation in the presence of acute coronary syndromes
	“New” acute HF
Criteria for urgent hospitalization	Severe liver distension, massive ascites or anasarca
	Decompensation in the presence of acutely decompensated noncardiac conditions, such as pulmonary disease or renal dysfunction
	Rapid and progressive onset of symptoms of HF
Consider hospitalization	Rapid drop in serum sodium (<130meq/L)
	Rapid elevation of creatinine (>2.5mg/dL)
	Symptoms persist at rest, despite optimized oral treatment
	Comorbidity with expected worsening of HF

Source: Bocchi et al^(1)^.

HF: heart failure.

## CLINICAL TREATMENT

### Non-pharmacological measures

Despite limited evidence, water and sodium restriction should be used in a customized fashion, and the daily weight should be used as a parameter of response to treatment.

### Monitoring and ventilatory support

Patients presenting with any sign of instability should be monitored by continuous electrocardiogram (ECG), noninvasive blood pressure and oximetry. Regardless of the form of presentation, hypoxia should be corrected in an attempt to ensure adequate oxygenation and reduce the respiratory work. Noninvasive ventilation (CPAP or BiPAP) resulted in a reduction of intubations and mortality, especially in acute pulmonary edema^(22)^.

### Vasodilators

Vasodilators act on the preload and afterload, requiring less myocardial consumptions than inotropic drugs. Retrospective studies have demonstrated lower mortality in DHF with the use of vasodilators^(23,24)^. They are indicated in situations of pulmonary and systemic congestion (profiles B and C) and in individuals with poor peripheral perfusion and SBP>90mmHg (profile C). The use of these agents requires intensive SBP monitoring and dose titration. In profile B patients who are asymptomatic at rest and with SBP>120mmHg, it is possible to use oral vasodilators and diuretics. Intravenous vasodilators should be used in patients with dyspnea at rest, and in acute pulmonary edema. These drugs should be avoided in patients with hypotension (SBP<90mmHg), hypovolemia and recent use of phosphodiesterase-5 inhibitors (sildenafil, vardenafil and tadalafil).

#### Nitroglycerin^(25)^

Nitroglycerin is a short-acting intravenous vasodilator. Small doses (30 to 40*μ*g/min) induce venodilatation, whereas higher doses (250*μ*g/min) cause arteriolar dilatation. Its benefits derive from venous dilatation, with relief in pulmonary congestion and increase in coronary flow, thus justifying its use in DHF associated with ACS. Headache and nausea are common side effects.

#### Sodium nitroprusside^(25)^

A potent arterial and venous vasodilator, sodium nitroprusside reduces the preload and afterload, thus improving the biventricular systolic performance. The usual dose is 0.5 to 10*μ*g/kg/min. It should be avoided in ACS because of the risk of decreasing the coronary perfusion pressure and “coronary steal”. Arterial hypotension is the most common side effect and may lead to hypoperfusion and worsening of the renal function.

Sudden discontinuation may cause a rebound effect. Thus, gradual withdrawal is advised, with the use of oral vasodilators. When high doses are used for a long period, especially in patients with renal and/or hepatic dysfunction, there is a risk of intoxication by thiocyanate and cyanide.

#### Inotropic agents^(26)^

In patients with low cardiac output, with or without congestion (profiles L and C), inotropic therapy may be required to improve tissue perfusion^(5)^. Although these drugs have been effectively used to increase perfusion and cardiac output, these hemodynamic parameters are not associated with better outcomes in patients with HF. They are associated with ischemia, and the intermittent use is not recommended. These agents are appropriate for short-term therapy in patients with hemodynamic deterioration, patients with chronic HF, increased levels of nitrogenous waste, and those who did not achieve satisfactory diuresis with diuretics and vasodilators. They are also efficient in the hemodynamic support of patients awaiting cardiac transplantation or revascularization, and may save lives in situations of cardiogenic shock. These drugs are not indicated in patients with HFPEF.

#### Dobutamine

Dobutamine is a beta-adrenergic agonist. It stimulates beta-adrenergic receptors 1 and 2, thus promoting elevation of adenyl cyclase and the subsequent increase in the intracellular calcium concentration, resulting in inotropism and chronotropism.

Its most common adverse effects are ischemia and arrhythmias, because of increased oxygen consumption.

Despite data suggesting increased mortality, dobutamine is the most widely used inotropic agent^(27)^. It provides hemodynamic improvement, with a dose-dependent increase in the cardiac output, and usually does not cause hypotension. It should be restricted to patients with DHF in profiles C and L, at a dose of 3 to 20*μ*g/kg/min. In hypotensive patients (SBP<70mmHg), combination with a vasopressor (dopamine or norepinephrine) may be considered.

#### Milrinone

Milrinone is a phosphodiesterase-III inhibitor. An inodilator agent for its inotropic and vasodilator properties (both systemic and pulmonary), it increases cardiac contractility and produces arterial and venous dilation by means of the increase in intracellular concentrations of cyclic AMP and calcium. It promotes an increase in cardiac output and reduction in the pulmonary and systemic vascular resistance.

A study has demonstrated increased mortality, especially in the ischemic etiology^(28)^. Because its mechanism of action does not depend on the adrenergic system, milrinone may be an option for patients using betablockers (aiming at maintaining them). Among its side effects, we should point out its arrhythmogenic potential (atrial and/or ventricular). The recommended dose ranges from 0.3 to 0.75*μ*g/kg/min. Due to the risk of hypotension, the loading dose is not recommended. In patients with renal failure, dose should be adjusted.

#### Levosimendan

Levosimendan is a calcium sensitizer. It exerts an inotropic action, increasing troponin C sensitivity to calcium that is already available in the cytoplasm, without additional calcium overload. It promotes contractile and hemodynamic improvement similar to other inotropic drugs and has a vasodilator action by activating ATPdependent potassium channels^(29)^. Levosimendan is safe and efficient in DHF of different etiologies, especially in patients using betablockers^(30,31)^. Its half-life is long, with active metabolites maintaining their effect for up to 7 days.

The major side effects include hypotension, headache, and arrhythmias (atrial and ventricular). The SURVIVE study^(32)^ compared levosimendan with dobutamine in patients eligible for inotropic support, and found no difference in the 180-day mortality. A maintenance dose of 0.1*μ*g/kg/min, in 24 hours, without a loading dose, has been suggested to reduce the side effects.

### Vasopressors

The most frequently used vasopressors are norepinephine and dopamine, which are indicated in symptomatic hypotension despite correction of volemia. Norepinephrine has a high affinity for alpha-adrenergic receptors and moderate affinity for beta-adrenergic receptors, with consequent and significant vasoconstriction, mild increase of the heart rate, inotropism and increased myocardial oxygen consumption. In DHF, it should be used in combination with other inotropic agents, for the treatment of cardiogenic shock refractory to other circulatory support measures. Dopamine also has beta and alpha-adrenergic effects, the latter at doses higher than 10*μ*g/kg/min – the usual dose ranges from 2 to 20*μ*g/kg/min. It is also associated with increased heart rate, myocardial oxygen consumption, myocardial ischemia, and ventricular arrhythmias^(33)^.

In DHF, discontinuation of betablockers remains controversial in the daily practice. In most of the patients with DHF (profile B – “wet-warm”), it is not necessary to discontinue or reduce betablockers. Despite the scarce literature available^(34,35)^, it seems clear that the abrupt withdrawal of betablockers may increase sympathetic activation even further (which is invariably already elevated in DHF), thus favoring apoptosis and arrhythmias, with a consequent reduction in survival. When inotropic drugs are necessary in patients with DHF previously using betablockers, the use of drugs that do not act in beta-adrenergic receptors such as milrinone and levosimendan may be considered. In patients with cardiogenic shock chronically using high doses of betablockers, the use of dobutamine is irrefutable, as well as the reduction and/or discontinuation of betablockers.

### Management of hypervolemia

#### Diuretics^(36)^

Diuretics reduce extracellular fluids, filling pressures and cardiac cavities, with a consequent improvement of performance, thus promoting fast symptomatic relief of congestion. They may be associated with adverse effects such as hypotension, abnormal serum electrolyte levels, renal dysfunction and neurohormonal activation by hypovolemia. The use of diuretics must be rational and judicious, aiming to preserve renal function (the lowest possible dose with the best result). The loop diuretics furosemide and bumetanide are the most indicated. In patients resistant to diuretics, continuous intravenous use is possible, as well as the combination with thyazide and aldosterone antagonists. In “new” acute HF diuretics should be carefully used, because some of these patients show normovolemia, or even hypovolemia^(37)^.

#### Hypertonic saline solution

A study with patients resistant to oral loop diuretics showed preservation and improvement of the renal function with the use of 150mL of NaCl solution at a concentration of 1.4 to 4.6% (according to the patient's serum sodium levels) followed by a high dose of intravenous furosemide, in comparison to the group which received intravenous furosemide alone^(38)^. Brazilian studies have also demonstrated preservation of the renal function and increased diuresis^(39,40)^. The profile of patients eligible for this procedure includes those with hyponatremia, systemic congestion (ascites and peripheral edema) and worsening of the renal function with the use of diuretics.

#### Ultrafiltration

This procedure is performed via a peripheral intravenous line, and can be potentially used in hypervolemic patients, those with frequent rehospitalizations, and on a day-hospital basis. A study has demonstrated reduction in weight and rehospitalizations within 90 days in comparison to the treatment with diuretics, despite a more pronounced initial elevation of creatinine^(41)^. A more recent trial has not shown any benefit with the use of this strategy^(42)^.

## SURGICAL TREATMENT

In patients with DHF secondary to ACS, coronary angiography is mandatory and may guide revascularization strategies (whether percutaneous or surgical). Likewise, acute valvular heart diseases may require percutaneous or surgical treatment^(7)^.

For patients with DHF, especially those with cardiogenic shock irresponsive to medical treatment with diuretics, vasodilators and inotropic drugs, the use of ventricular assist devices (VAD) may be considered. These may classified as short or long-term devices. Short-term devices include intra-aortic baloon pump, ECMO, impella^TM^, transcore^TM^, rota-flow^TM^ and centrimag^TM^; they may be used as bridge to recovery (cardiogenic shock in ACS, post-cardiotomy and myocarditis), bridge to decision (post-cardiopulmonary arrest), and bridge to bridge (stabilization and implantation of a long-term VAD). The long-term devices or artificial ventricles (paracorporeal or implantable) are considered as bridge to transplantation or as destination therapy (when transplantation is contraindicated)^(43)^.

Patients with HF refractory to medical treatment and frequent hospitalizations for DHF may be considered eligible for surgical treatment including VAD and cardiac transplantation^(44)^.

## CONCLUSION

DHF is a frequent cause of hospitalization and has a high risk of rehospitalization and mortality. From the diagnosis and risk stratification of DHF, determination of the clinical hemodynamic profile is fundamental to guide therapy including non-pharmacological and pharmacological measures, and, in refractory cases, VAD and cardiac transplantation.
